# Parkinson's Disease Cell Transplantation Therapy: A New Dawn With Pluripotent Stem Cell–Based Therapy

**DOI:** 10.1111/jnc.70500

**Published:** 2026-07-01

**Authors:** Etsuro Nakanishi, Hodaka Yamakado, Nobukatsu Sawamoto, Jun Takahashi, Ryosuke Takahashi

**Affiliations:** ^1^ Department of Neurology Kyoto University Graduate School of Medicine Kyoto Japan; ^2^ Department of Clinical Application Center for iPS Cell Research and Application (CiRA) Kyoto Japan; ^3^ Kyoto University Office of Research Acceleration Kyoto Japan

**Keywords:** cell transplantation therapy, embryonic stem cell, fetal ventral mesencephalic, induced pluripotent stem cell, Kyoto trial, Parkinson's disease

## Abstract

Parkinson's disease (PD), a prevalent neurodegenerative disorder, is characterized by progressive loss of dopaminergic neurons in the midbrain. While dopamine replacement therapy effectively manages early symptoms, its long‐term use leads to motor complications, highlighting the urgent need for treatments that directly address the underlying pathological changes. Cell transplantation, which aims to replace the lost dopaminergic neurons, has emerged as a promising approach. Early attempts using fetal ventral mesencephalic (fVM) tissue showed proof‐of‐concept, with some patients experiencing long‐term motor improvement. However, these trials have been hampered by inconsistent results, graft‐induced dyskinesia (GID), and significant ethical and logistical issues related to tissue supply. These challenges have shifted the focus to pluripotent stem cells (PSCs), including human‐induced pluripotent stem cells (iPSCs) and embryonic stem cells (ESCs), which offer a stable, ethically sound, and scalable source of high‐quality cells. Recent clinical trials using PSCs suggest a turning point. All reported clinical trials demonstrated the safety and feasibility of this approach. The need for long‐term safety and efficacy data, patient stratification, and techniques to improve graft survival are key areas of future research. Nevertheless, recent clinical trial successes suggest that cell transplantation is moving beyond symptomatic relief to become a truly restorative therapy for PD.

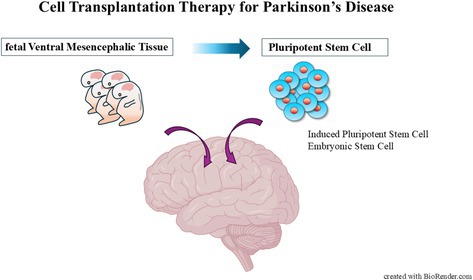

## Graphical abstract

Parkinson's disease (PD) is characterized by the progressive loss of dopaminergic neurons in the midbrain. Cell transplantation, which aims to replace these lost neurons, has emerged as a promising approach. Early attempts using fetal ventral mesencephalic tissue showed proof‐of‐concept, with some patients achieving long‐term motor improvement. However, limitations associated with this approach have led to a shift toward pluripotent stem cells (PSCs). Recent clinical trial results using PSC‐derived cells suggest a paradigm shift—moving beyond conventional symptomatic management toward a new era of regenerative medicine aimed at restoring neurological function in patients with PD.

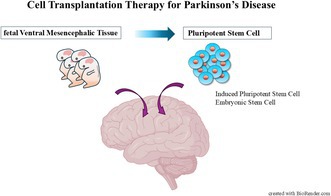

**Abbreviations**
AE: adverse event
DRT: dopamine replacement therapy
ESC: embryonic stem cell
fVM: fetal ventral mesencephalic
GDNF: glial cell line–derived neurotrophic factor
GID: graft‐induced dyskinesia
HLA: human leukocyte antigen
iPSC: induced pluripotent stem cell
MSC: mesenchymal stromal cell
PD: Parkinson's disease
PET: positron emission tomography
PSC: pluripotent stem cell
QoL: quality of life
SAE: serious adverse event

## 1 Introduction

Parkinson's disease (PD) is one of the most prevalent neurodegenerative disorders, and its prevalence is increasing worldwide (Poewe et al. 2017; Dorsey et al. 2018; Tanner and Ostrem 2024). The pathological hallmarks of PD are abnormal aggregation of α‐synuclein and the formation of intracellular inclusions known as Lewy bodies, accompanied by progressive degeneration and loss of dopaminergic neurons in the substantia nigra pars compacta. This degeneration leads to a marked depletion of dopamine in the striatum, resulting in characteristic motor manifestations of the disease, including bradykinesia or akinesia, rigidity, and resting tremor.

Current treatment for PD is centered on dopamine replacement therapy (DRT), primarily levodopa (Poewe et al. 2017; Armstrong and Okun 2020; Tanner and Ostrem 2024). DRT demonstrates robust efficacy in the early stages of the disease and produces a dramatic improvement in patients' quality of life (QoL). However, as the disease progresses, motor complications such as wearing‐off phenomena and dyskinesias emerge, making symptom control increasingly challenging and leading to a decline in QoL (Armstrong and Okun 2020; Tanner and Ostrem 2024). Thus, increasing difficulty in controlling symptoms as the disease advances underscores the limitations of current pharmacological therapies and has created a strong impetus to develop therapies that can halt disease progression or restore lost neuronal function.

To address these unmet needs, cell transplantation therapy has been extensively explored as a regenerative approach. This method aims to replenish lost dopaminergic neurons and reconstitute dopaminergic innervation within the striatum (Barker et al. 2015, 2024). Cell transplantation therapy for PD directly addresses the fundamental pathology of dopaminergic neuron loss, a target that cannot be adequately addressed by pharmacological therapy or surgical approaches such as deep brain stimulation (DBS). Early efforts in this field advanced through the transplantation of fetal ventral mesencephalic (fVM) tissue obtained from elective abortions, while more recently, transplantation of pluripotent stem cell (PSC)–derived neurons has emerged as a reliable and increasingly mature therapeutic strategy (Lindvall et al. 1989; Barker et al. 2015, 2024; Sawamoto et al. 2025; Tabar et al. 2025). PSCs provide a stable and renewable source of cells for transplantation, and induced pluripotent stem cells (iPSCs) in particular have attracted attention due to fewer minimal ethical concerns (Takahashi et al. 2025). Recent clinical trials using PSC‐derived grafts have provided evidence supporting the potential of this approach for future therapies (Sawamoto et al. 2025; Tabar et al. 2025).

In this review, we trace the historical development of fVM transplantation therapy and highlight the advances achieved with PSC–derived dopaminergic progenitor cells, including human‐iPSCs and embryonic stem cells (ESCs). We focus on recent clinical outcomes of PSC‐based transplantation and provide a discussion of the remaining challenges and future perspectives.

## 2 Evolution of Cell Transplantation Therapy for Parkinson's Disease

**FIGURE 1:**
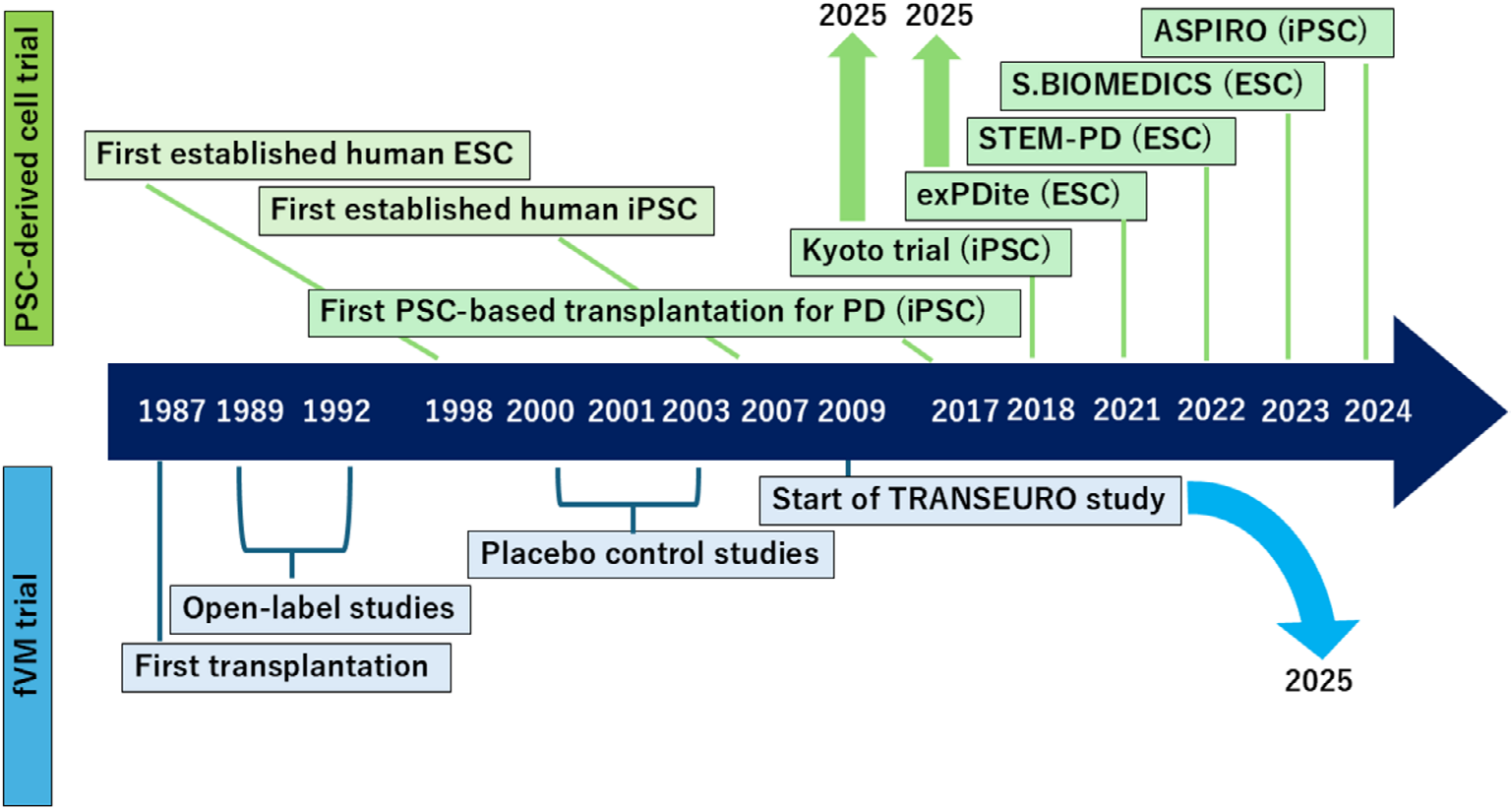
Evolution of clinical research on transplantion therapy using fetal ventral mesencephalic and pluripotent stem cells. ESC, embryonic stem cell; fVM, fetal ventral mesencephalic; iPSC, induced pluripotent stem cell.

Cell transplantation therapy for PD began in the early 1980s, with adrenal medullary autografting performed at the Karolinska Institute in Sweden (Björklund and Lindvall 2017). It subsequently advanced in the late 1980s through pioneering efforts at Lund University, where tissue from aborted fVM was transplanted into two patients (Figure 1) (Backlund et al. 1985; Lindvall et al. 1989). The fVM contains dopaminergic progenitor cells that, when transplanted into the striatum, were expected to mature into dopaminergic neurons, thereby ameliorating parkinsonian symptoms (Björklund et al. 1980; Barker et al. 2015, 2024). In early open‐label trials, some patients exhibited sustained motor improvement lasting several years after transplantation, and ^18^F‐DOPA positron emission tomography (PET) imaging provided objective evidence that the grafts survived in the brain and produced dopamine, thereby establishing proof of concept (Lindvall et al. 1990; Barker et al. 2015, 2024). These findings suggested that cell transplantation could serve as a disease‐modifying therapy for PD, thereby generating considerable enthusiasm. However, ethical concerns regarding the use of fetal tissue and difficulties in securing a stable supply were also identified as major obstacles to clinical implementation. Furthermore, cellular heterogeneity within grafts was recognized as a critical issue. In particular, graft‐induced dyskinesia (GID), potentially mediated by the inclusion of serotonergic neurons, was reported as a clinically significant adverse event (Freed et al. 2001; Olanow et al. 2003; Lane et al. 2010; Barker et al. 2013). Moreover, double‐blind trials conducted in the 1990s failed to replicate the anticipated efficacy, highlighting variability in therapeutic outcomes as well as the persistent challenge of GID. Subsequently, the TRANSEURO trial (NCT01898390)—a study designed to re‐evaluate the efficacy of fVM transplantation using optimized protocols—highlighted the persistent challenge of securing sufficient fetal tissue (Lane et al. 2010; Politis et al. 2011; Barker et al. 2013, 2024, 2025; Barker and the TRANSEURO Consortium 2019). In addition, non‐severe GID was observed in patients exhibiting high uptake of the serotonin transporter ligand (^11^C‐DASB) on PET imaging. This finding is consistent with the prevailing hypothesis implicating serotonergic neurons in the development of GID (Barker et al. 2025).

To overcome the inherent limitations of fetal tissue transplantation, cell‐based therapies leveraging PSCs—namely ESCs and iPSCs—have emerged as a promising strategy. PSCs offer significant advantages owing to their theoretical capacity for unlimited self‐renewal, thereby enabling the scalable production of homogeneous and standardized cellular products. In transplantation therapy for PD, directed differentiation into A9‐subtype dopaminergic neurons, characteristic of the substantia nigra, is considered particularly critical for functional recovery (Grealish et al. 2010). Established differentiation protocols typically involve neural induction via dual‐SMAD inhibition, followed by ventralization through Sonic hedgehog (SHH) signaling, in combination with activation of FGF8 and WNT pathways (e.g., via GSK‐3β inhibition), thereby directing regional specification toward midbrain dopaminergic lineages (Chambers et al. 2009; Doi et al. 2014). Furthermore, cell‐sorting strategies using surface markers such as CORIN enable enrichment of target cell populations while minimizing contamination by off‐target cells—notably serotonergic neurons implicated in GID—as well as undifferentiated cells with tumorigenic potential (Doi et al. 2014, 2020; Kikuchi et al. 2017). Collectively, PSC‐derived therapies provide a promising technological platform that addresses the key limitations of human fetal ventral mesencephalic (hfVM) transplantation, including supply instability, ethical constraints, and variability in cell composition. By enabling the scalable and reliable production of high‐quality, uniform cellular products, this approach represents an advance toward clinical application.

## 3 Recent Clinical Trials Using Pluripotent Stem Cells

Currently, clinical trials employing PSC–derived grafts, most prominently those generated from ESCs and iPSCs, are underway worldwide for the treatment of PD and are attracting considerable attention (Barker et al. 2024; Kirkeby et al. 2025; Takahashi et al. 2025; Chang et al. 2025) (Table 1; Figure 1). In 2025, several phase I or phase I/II clinical trials utilizing iPSC‐ or ESC‐derived dopaminergic progenitor cells reported primary results or interim analyzes (Sawamoto et al. 2025; Tabar et al. 2025; Chang et al. 2025). Across these studies, safety was consistently demonstrated, and findings suggested potential efficacy (Table 2) (Sawamoto et al. 2025; Tabar et al. 2025; Chang et al. 2025). The following sections summarize the study designs and key outcomes of each trial.

**TABLE 1:** Summary of pluripotent stem cell transplantation trial for PD.

Trials	Type of cell	Immunosuppressant status (yes/no)	Clinical trial phase	Start year	Location
Kyoto trial	Allogeneic iPSC	Yes	I/II	Since 2018 (Completed)	Japan
exPDite	ESC	Yes	I	Since2021 (Complete)	USA/Canada
STEM‐PD	ESC	Yes	I	Since 2022	Sweden/UK
S.BIOMEDICS	ESC	Yes	I/II	Since 2023	South Korea
ASPIRO	Autologous iPSC	No	I	Since 2024	USA

Abbreviations: ESC, embryonic stem cell; iPSC, induced pluripotent stem cell.

**TABLE 2:** Comparative summary of recently reported outcomes of cell transplantation therapies in PD.

	Kyoto trial	exPDite	S.BIOMEDICS^a^	TRANSEURO
**Trial characteristics**				
Study Design	Single‐, open‐label, single‐arm, phase I/II trial	Multicenter, open‐label, single‐arm, phase I trial	Single‐center, open‐label, single‐arm, phase I/IIa trial	Multicenter, randomized, open‐label, controlled Trial
Cell source	Allogeneic iPSCs	ESCs	ESCs	fMV
Cell dose	2.1–2.6 million cells per putamen (low dose); 5.3–5.5 million cells per putamen (high dose)	0.9 million cells per putamen (low dose); 2.7 million cells per putamen (high dose)	3.15 million cells per putamen (low dose); 6.3 million cells per putamen (high dose)	three fVMs per putamen
Immunosuppression regimen	Tacrolimus	Basiliximab, steroid, tacrolimus	Basiliximab, steroid, tacrolimus	Cyclosporin, azathioprine, steroid
Transplantation site	dorsal and caudal putamen	post‐commissural putamen	bilateral whole putamen	putamen
Efficacy assessment timepoints (months)	24	18	12^a^	36
**Baseline characteristics**				
Number of patients	Total 7^b^ (low 3, high 4)	Total 12 (low 5, heigh 7)	Total 12 (low 6, high 6)	11 patients were grafted^c^
Age (years)	Total 60.0 (50, 69)	Total 67.0 (64.5, 70.0)	Total 60.3 (5.0)	51.8 (9.2)
	Low 60.0 (50, 62)	Low 67.0 (65.0, 67.0)	Low 60.0 (5.9)	
	High 59.5 (56, 69)	High 68.0 (64.0, 70.0)	High 60.7 (4.6)	
Disease Duration (years)	Total 9.9 (1.2)	Total 9.0 (5.9, 11.5)	Total 10.5 (2.5)	N.A.
	Low 9.6 (0.9)	Low 12.7 (8.9, 13.7)	Low 9.2 (2.7)	
	High 10.2 (1.5)	High 8.2 (5.3, 10.2)	High 11.8 (1.6)	
MDS‐UPDRS Part III (OFF state)	Total 50.8 (14.6)	Total 48.5 (39.0, 53.0)	Total 59.3 (8.0)	31 (9.2)
	Low 44.5 (14.8)	Low 44.0 (34.0, 50.0)	Low 61.0 (9.1)	
	High 54.0 (15.5)	High 51.0 (42.0, 11.2)	High 57.7 (7.1)	
MDS‐UPDRS Part III (ON state)	Total 17.7 (8.3)	Total 23.0 (16.0, 32.0)	Total 26.5 (3.9)	20 (10.9)
	Low 10.5 (3.5)	Low 18.0 (15.0, 23.0)	Low 27.5 (3.0)	
	High 21.3 (7.7)	High 26.0 (17.0, 34.0)	High 25.5 (4.7)	
Baseline LEDD (mg)	Total 1125.48 (242.30)	Total 1236 (603)	Total 1565.79 (456.15)	516.5 (360.1)
	Low 1047.75 (127.49)	Low 1559 (614)	Low 1277.92 (373.99)	
	High 1164.35 (293.91)	high 1006 (516)	High 1853.67 (344.95)	
**Clinical outcome**				
Change from baseline in MDS‐UPDRS Part III (OFF state)	Total –9.5 (13.8)	Total –17.0 (20.0)	Total –14.1 (6.2)	No significant difference vs. control
	Low −8.0 (4.2)	Low −8.6 (29.2)	Low −12.7 (8.2)	
	High −10.3 (17.6)	High −23 (7.9)	High −15.5 (3.6)	
**Limitations**				
Limitations	Small sample size; open‐label, single‐arm design without sham or control group	Small sample size; open‐label, single‐arm design without sham or control group	Small sample size; open‐label, single‐arm design without sham or control group	Small sample size due to tissue availability issues

*Note:* These values are described either the mean (standard deviation) or the median (Q1, Q3).

Abbreviations: ESC, embryonic Stem Cell; fMV, fetal ventral mesencephalon; iPSC, induced pluripotent stem cell; LEDD, levodopa equivalent daily dose.

^a^ The summary of the S.BIOMEDICS trial is derived from an interim analysis with follow‐up of up to 12 months.

^b^ The first patient underwent staged unilateral surgeries with an eight‐month interval and was included only in the safety assessment.

^c^ A single case underwent a unilateral surgery because did not wish to proceed to a second graft because of the unpredictability of surgical planning and the associated psychological stress.

### 3.1 Phase I/II Trial of Allogeneic iPSC‐Derived Dopaminergic Cells for PD (Kyoto Trial)

The iPSC‐based clinical trial conducted by our team at Kyoto University, Japan (jRCT2090220384; hereafter referred to as the Kyoto trial), the results of which were recently reported (Sawamoto et al. 2025), was a single‐center, single‐arm, open‐label phase I/II study. This trial was designed to evaluate the safety and preliminary efficacy of striatal transplantation of allogeneic iPSC‐derived dopaminergic progenitors in PD patients. The transplanted cells were produced from a rigorously quality‐controlled iPSC stock established from a healthy donor, following the differentiation protocol developed by Takahashi et al. (Doi et al. 2014, 2020; Kikuchi et al. 2017; Takahshi 2020; Sawamoto et al. 2025). In this phase I/II clinical trial, seven patients with PD received bilateral transplantation of iPSC‐derived dopaminergic progenitor cells. A total of 7 participants were assigned to either a low‐dose cohort (2.1–2.6 million cells per putamen, *n* = 3) or a high‐dose cohort (5.3–5.5 million cells per putamen, *n* = 4). Tacrolimus monotherapy was administered for immunosuppression for 15 months following transplantation (Sawamoto et al. 2025). The primary endpoint was safety, while preliminary efficacy was assessed as a secondary endpoint in six participants who underwent one‐stage bilateral grafting.

The principal findings of the Kyoto trial established the safety of the procedure. Apart from a single case of controllable moderate dyskinesia, only mild adverse effects were reported. The dyskinesia observed as a moderate adverse event was clinically distinguishable from GID and remained manageable with dose reduction of anti‐parkinsonian medications. This suggests that the dyskinesia was likely related to increased striatal dopamine levels following transplantation. Notably, no GID, a complication previously reported in fVM transplantation studies, was observed. Furthermore, no evidence of tumor formation, as assessed by MRI and ^18^F‐FLT PET, was observed up to 24 months post‐transplantation. Furthermore, MDS‐UPDRS Part III OFF scores improved in 4 of the 6 evaluable participants, with a mean reduction of −9.5 points (Sawamoto et al. 2025). Notably, three participants achieved ≥ 30% improvement in OFF scores, a threshold commonly considered indicative of a clinically meaningful response to oral dopaminergic therapy. ^18^F‐DOPA PET imaging demonstrated an increase in striatal dopamine uptake (Ki value), an index of dopaminergic terminal function, 24 months post‐transplantation, with a more pronounced change in the high‐dose group (Sawamoto et al. 2025).

### 3.2 Phase I Trial of ESC‐Derived Dopaminergic Cells for PD (eXPDite)

This phase I, multicenter, single‐arm, open‐label clinical trial (NCT04802733) evaluated bemdaneprocel, an ESC–derived dopaminergic progenitor product, and was conducted in the United States and Canada (Tabar et al. 2025). A total of 12 patients were enrolled and assigned to either a low‐dose group (0.9 million cells per putamen, *n* = 5) or a high‐dose group (2.7 million cells per putamen, *n* = 7). The immunosuppressive regimen consisted of intravenous basiliximab and methylprednisolone, followed by oral prednisolone (5 mg/day) and tacrolimus for 12 months postoperatively (Tabar et al. 2025). Serious adverse events (SAEs) included COVID‐19 infection and surgery‐related seizures; however, the latter resolved with antiepileptic treatment for two months, with no recurrence after discontinuation. Brain MRI at 18 months post‐transplantation revealed no evidence of tumor formation (Tabar et al. 2025), indicating an acceptable safety profile. Notably, dyskinesia was reported as an adverse event in two patients in the low‐dose group, with no GID observed, consistent with findings from the Kyoto trial. Regarding efficacy, improvements in the MDS‐UPDRS Part III OFF score were observed in three of five patients in the low‐dose group, with a mean reduction of −8.6 points from baseline, whereas all patients in the high‐dose group showed improvement, with a mean reduction of −23.0 points (Tabar et al. 2025). Furthermore, mean ^18^F‐DOPA PET uptake in the putamen increased in both groups (Tabar et al. 2025).

### 3.3 Phase I/IIa Trial of ESC‐Derived Dopaminergic Cells for PD (S.BIOMEDICS)

This phase I/IIa, single‐center, single‐arm, open‐label clinical trial (NCT05887466), conducted by S.BIOMEDICS in South Korea, evaluated human embryonic stem (hESC)–derived dopaminergic progenitors (A9‐DPC) (Chang et al. 2025). Employing a standard 3 + 3 dose‐escalation design, 12 patients were assigned to either a low‐dose group (3.15 million cells per putamen, *n* = 6) or a high‐dose group (6.30 million cells per putamen, *n* = 6) (Chang et al. 2025). Recently, 12‐month post‐transplantation outcomes were reported. The immunosuppressive regimen included intravenous basiliximab and methylprednisolone, followed by oral prednisolone (5 mg/day) and tacrolimus for 12 months (Chang et al. 2025). Adverse events (AEs) included one case of surgery‐related intracerebral hemorrhage and three events considered secondary to immunosuppression (transient hyperkalemia, idiopathic thrombocytopenia, and new‐onset diabetes mellitus). Notably, no AEs were attributed to the A9‐DPC graft (Chang et al. 2025). Furthermore, brain MRI and ^18^F‐FDG PET revealed no evidence of abnormal cell proliferation or tumor formation (Chang et al. 2025). Regarding efficacy, 11 of 12 patients showed improvement in the MDS‐UPDRS Part III OFF score, with only one patient in the low‐dose group failing to respond. The mean reduction from baseline was −12.7 points in the low‐dose group, whereas all patients in the high‐dose group achieved clinical improvement, with a mean reduction of −15.5 points (Chang et al. 2025). Additionally, ^18^F‐FP‐CIT uptake in the posterior putamen increased in both groups, with more pronounced increases observed in the high‐dose group (Chang et al. 2025).

## 4 Mechanisms of Action of PSC‐Derived Cell Therapy in PD

Cell transplantation therapy is a cell‐based dopamine replacement strategy predicated on the understanding that striatal dopamine depletion represents a primary pathophysiological substrate underlying the motor symptoms of PD. For therapeutic efficacy to be achieved, transplanted cells must successfully engraft, differentiate into functional dopaminergic neurons, and establish appropriate synaptic integration within host neural circuits.

Although definitive evidence demonstrating reconstruction of a physiological nigrostriatal circuit remains limited, postmortem histological analyzes have demonstrated that, in patients who experienced clinical improvement following hfVM transplantation, grafted cells survived long term and maintained dopaminergic reinnervation (Li et al. 2016). Similarly, stem cell–based studies in rodent and non‐human primate models have shown that engrafted and differentiated cells can restore motor function through dopaminergic reinnervation (Doi et al. 2014, 2020; Kikuchi et al. 2017). Furthermore, a study in rat models has revealed that these transplanted neurons receive synaptic inputs from host neurons, indicating their functional integration into the host neural circuitry (Adler et al. 2019). These findings support the expectation that similar therapeutic mechanisms may operate in humans.

Functional reconstruction mediated by transplanted cells in vivo is primarily assessed using nuclear imaging as surrogate markers. Common approaches utilize radioligands for L‐DOPA and the dopamine transporter (DAT). The former, typically assessed by ^18^F‐DOPA PET, reflects dopamine synthesis capacity mediated by aromatic L‐amino acid decarboxylase (AADC), whereas the latter (e.g., ^18^F‐FP‐CIT PET) serves as a proxy for presynaptic terminal density. While these measures cannot inherently distinguish between graft‐ and host‐derived dopaminergic terminals, studies of hfVM transplantation have demonstrated that increased ^18^F‐DOPA uptake reflects graft survival and dopaminergic reinnervation (Kordower et al. 1995, 1998). Furthermore, improvements in ^18^F‐DOPA uptake correlate with clinical motor recovery for several years after transplantation (Ma et al. 2010).

These findings suggest that therapeutic efficacy may depend on the final yield of engrafted dopaminergic neurons. In non‐human primate models, only approximately 1%–2% of transplanted cells are reported to survive and differentiate into dopaminergic neurons (Comini and Dowd 2025). Given that the healthy human substantia nigra contains approximately 300 000 dopaminergic neurons, and that approximately 100 000 neurons are estimated to be required for symptomatic improvement (Rudow et al. 2008; Hagell and Brundin 2001), a first‐order approximation—accounting for low engraftment efficiency—suggests that between 5 and 10 million cells per putamen may be required. In the Kyoto trial, the cell dose administered to the high‐dose group was near the lower bound of this estimated range. Consistent with this estimation, while ^18^F‐DOPA Ki values increased in a dose‐dependent manner, the magnitude of improvement—even in the high‐dose group—remained close to the threshold associated with symptomatic improvement (Sawamoto et al. 2025). These findings suggest that further strategies to increase the number of engrafted cells warrant investigation. Moreover, a modest correlation between ^18^F‐DOPA Ki values and MDS‐UPDRS Part III (OFF) scores was observed at the group level; however, this relationship was not consistently evident at the individual level. This discrepancy underscores the mechanistic complexity of cell replacement therapy. Clinical outcomes in PD are likely influenced by non‐dopaminergic systems (e.g., cholinergic and noradrenergic pathways) as well as broader network plasticity (Popescu et al. 2024). Furthermore, ^18^F‐DOPA uptake does not directly reflect postsynaptic neuronal activity. Consequently, reliance on a single imaging biomarker may be insufficient to fully account for clinical efficacy.

Besides, the lower engraftment efficiency observed in non‐human primates compared with rodents suggests that clinical translation to humans may face even greater challenges in terms of cell survival (Comini and Dowd 2025). Consistent with this notion, recent trials by exPDite and S.BIOMEDICS have indicated a trend toward greater efficacy in higher‐dose cohorts (Tabar et al. 2025; Chang et al. 2025). In addition, factors such as the maturation state of the graft, the anatomical distribution of transplanted cells, and interactions with the host environment are critical determinants of functional recovery. Taken together, the optimal cell dose and delivery strategies remain to be fully established.

## 5 Current Status and Future Perspectives of Immunosuppressive Therapy in Cell Transplantation for PD

Immune rejection remains a critical challenge in cell transplantation therapy. Such rejection can markedly reduce graft survival, thereby diminishing the therapeutic efficacy. The concept of the brain as an “immune‐privileged” site adds further complexity to this issue. Many ongoing trials continue to adopt prolonged immunosuppressive therapy to prevent graft rejection. However, long‐term multi‐agent immunosuppression imposes a substantial burden on patients, resulting in increased risks of infection, as well as adverse effects such as renal impairment and metabolic disturbances. Indeed, a death due to an opportunistic infection has been reported in a clinical trial (STEM‐PD, 2024) and was likely related to the immunosuppressive regimen. Conversely, inadequate immune control has been associated with graft rejection after discontinuation of immunosuppressive agents, as previously reported (Olanow et al. 2003).

Several strategies have been proposed for iPSC‐based approaches to address this immunological challenge. One option is autologous transplantation, in which iPSCs are generated from the patient's own somatic cells and subsequently differentiated for grafting (Schweitzer et al. 2020). Clinical trials investigating autologous iPSC‐derived cell transplantation are currently underway, and their results are awaited (Takahashi et al. 2025). Although this strategy is expected to virtually eliminate the risk of immune rejection, it presents substantial obstacles in terms of the time and cost required for individualized cell preparation. In addition, in autologous transplantation, grafted cells derived from patients with genetic susceptibility will inherently share the same genetic background and associated vulnerabilities as the host.

Another strategy involves generating iPSCs from donors who are homozygous for high‐frequency human leukocyte antigen (HLA) haplotypes and establishing a pre‐existing iPSC stock. This is one of the major advantages of iPSCs derived from somatic cells. In the Kyoto trial, transplanted cells were drawn from the national “iPSC stock project” and carried the HLA haplotype most prevalent in the Japanese population in a homozygous state (Okita et al. 2011). Building on preclinical findings, the Kyoto trial employed a relatively mild immunosuppressive regimen consisting of tacrolimus monotherapy even for recipients without HLA matching (Kikuchi et al. 2017; Sawamoto et al. 2025). In the Kyoto trial, no production of graft‐specific antibodies was detected, and the transplanted cells engrafted successfully, with no clinically significant immune reactions observed (Morizane et al. 2025). However, mixed lymphocyte reaction (MLR) assays demonstrated higher reactivity in HLA‐mismatched cases; although these responses were not clearly associated with clinical outcomes, they suggest that HLA mismatch may confer a risk of subclinical immune activation (Morizane et al. 2025).

These findings further suggest that, over the long term, such immune activation may compromise graft survival through chronic low‐grade inflammation or delayed immune responses, including chronic rejection. Accordingly, the development of sensitive and specific monitoring strategies capable of detecting these immunological changes represents an important research priority for optimizing post‐transplant management and immunosuppressive therapy. In particular, the establishment of non‐invasive, longitudinal assessment platforms—such as those based on fluid biomarkers and molecular imaging—is warranted.

While iPSC stocks represent a promising strategy, they possess inherent limitations. Even in relatively genetically homogeneous populations, such as that of Japan, achieving comprehensive coverage of HLA diversity remains challenging. In addition, constructing iPSC stock systems applicable to global populations would be even more difficult from both cost and logistical perspectives. Importantly, the Kyoto trial utilized an iPSC line (QHJI01s04) homozygous for the most prevalent HLA haplotype in the Japanese population (Okita et al. 2011), allowing direct comparison of immune responses under tacrolimus treatment between HLA‐matched and HLA‐mismatched conditions. Notably, no significant clinical differences were observed between these groups, and immune rejection was adequately controlled (Sawamoto et al. 2025). These findings suggest that the therapeutic outcomes of the Kyoto trial may be applicable beyond HLA‐matched settings, including to HLA‐mismatched, non‐Japanese populations. However, caution is warranted in generalizing these findings, as they were obtained from a single iPSC line derived from an individual with a specific HLA haplotype. It cannot be excluded that this specific HLA genotype or cell line possesses intrinsic characteristics that render it less prone to eliciting robust immune responses than other iPSC lines. Consistent with the potential broader applicability of this approach, clinical trials using the same iPSC‐derived product (“rangneprocel”) are currently underway in the United States.

To address the projected global increase in PD, often described as a “pandemic,” the development of scalable, quality‐controlled, off‐the‐shelf cell products is essential. In this context, genetically engineered universal donor iPSCs designed to minimize immunogenicity have emerged as a promising alternative approach (Deuse et al. 2019; Kim et al. 2025). Current approaches include modulation of HLA class I and II expression, as well as the incorporation of immune‐evasive mechanisms to evade host immune surveillance. If successfully established, these technologies may partially or completely obviate the need for long‐term immunosuppressive therapy, representing a substantial advance in both safety and clinical feasibility.

## 6 Remaining Challenges and Future Perspectives

Despite recent clinical milestones, several critical challenges remain to be addressed before stem cell–based transplantation can be established as a standard therapy. A primary concern is the enhancement of graft survival. Given the strong correlation between the number of engrafted cells and clinical efficacy, the currently low survival rate—estimated at approximately 1%–2%—remains a major limiting factor. Although increasing the total number of transplanted cells represents the most straightforward approach, this strategy may not be optimal because it carries several significant risks. Beyond the risk of localized mechanical injury to host tissue associated with larger injection volumes, transplanting a larger number of cells may also increase the likelihood of introducing undifferentiated stem cells or proliferating neural progenitor cells (Doi et al. 2014). Furthermore, the poorly vascularized and diffusion‐limited microenvironment present during the early post‐transplantation period may preferentially confer a survival advantage to immature cells that rely predominantly on glycolysis rather than oxidative phosphorylation for energy production (McMurtrey 2017; Hakami et al. 2025). This environment may therefore exert a selective pressure favoring undifferentiated cells with proliferative and potentially tumorigenic properties, which could ultimately contribute to excessive graft expansion over time. To mitigate the risk of contamination by such undesirable cells, the Kyoto trial successfully employed a cell purification strategy based on sorting for CORIN, a cell‐surface marker enriched in midbrain dopaminergic progenitors (Kikuchi et al. 2017; Sawamoto et al. 2025). Nevertheless, directly improving the intrinsic survival of the target grafted cells remains an important challenge for further optimizing cell replacement therapy. Pharmacological and biological interventions are being explored to address this challenge. For instance, zonisamide, an agent with both antiepileptic and anti‐parkinsonian properties, has been reported to enhance graft survival (Miyawaki et al. 2020). In addition, a deficiency of neurotrophic factors in the host microenvironment has been shown to compromise graft viability (Brundin et al. 2000; Rodríguez‐Pallares et al. 2023), underscoring the importance of optimizing the transplantation niche. In this context, strategies such as the administration of glial cell line–derived neurotrophic factor (GDNF) and the co‐transplantation of mesenchymal stromal cells (MSCs) have demonstrated potential for improving graft survival (Moriarty et al. 2017; Gantner et al. 2020; Rodríguez‐Pallares et al. 2023).

In addition to biological factors, mechanical injury during the transplantation procedure and the associated inflammatory response represent important determinants of cell survival. Recent studies have demonstrated that surgical manipulation, particularly needle trauma, can trigger acute inflammation and that controlling this response markedly improves graft survival (Park et al. 2023). In contrast, the TRANSEURO trial suggested that inter‐center variability in surgical instrumentation and transplantation techniques may influence transplantation outcomes (Barker et al. 2025), underscoring the importance of standardized and optimized delivery devices. Furthermore, optimization of surgical techniques, including the selection of transplantation sites, remains an open question. It is unclear whether broader distribution of transplanted cells within the striatum yields superior functional recovery compared with more localized delivery to the dorsal putamen (Table 2). Taken together, a multifaceted approach integrating biological, pharmacological, and surgical refinements will be essential for the future success of PSC‐based therapies.

Second, it is crucial to precisely characterize clinical determinants—such as patient demographics and disease stage—to identify those most likely to benefit from transplantation. Insights from hfVM transplantation suggest that younger age (< 60 years) and milder motor impairment (MDS‐UPDRS Part III < 50) are predictive of favorable outcomes (Freed et al. 2001; Olanow et al. 2003). In the Kyoto trial, although the most pronounced therapeutic effect was observed in the youngest participant and the most limited benefit in the oldest, age alone did not fully account for treatment response (Sawamoto et al. 2025). Similarly, disease duration did not emerge as a definitive predictive factor. Regarding baseline motor severity, patients with better outcomes tended to have lower MDS‐UPDRS Part III scores in both ON and OFF states. In particular, the ON‐state Part III score is considered a proxy for postsynaptic and non‐dopaminergic network function; however, findings across clinical trials remain inconsistent, and its validity as a predictive biomarker requires further investigation. In contrast, the baseline OFF‐state score appears more consistently associated with treatment response, with data from the S.BIOMEDICS trial also suggesting that patients with milder symptoms may derive greater benefit (Chang et al. 2025).

At present, the cumulative number of patients undergoing stem cell transplantation remains limited, posing a challenge for the identification of reliable biomarkers for patient selection. However, in PD, substantial progress has been made in the development of fluid and neuroimaging biomarkers, as well as biomarker‐based staging systems and prognostic biomarkers (Vijiaratnam and Foltynie 2023; Sakato et al. 2024; Simuni et al. 2024). With further advances in cell transplantation therapies, it is anticipated that candidate predictive biomarkers will emerge from these modalities. The integration of these complementary approaches may further refine patient stratification. Future clinical trials should incorporate stratification strategies and prospective analyzes to better elucidate these factors. Moreover, key limitations of existing studies include their open‐label design without sham controls, the absence of concurrent control groups, and small sample sizes. In Japan, placebo‐controlled designs for surgical interventions remain ethically and institutionally challenging. Accordingly, rigorous methodological approaches are essential to minimize bias, including blinded assessments, the use of objective endpoints, and the incorporation of external control cohorts where feasible.

Third, long‐term evaluation of safety and efficacy is imperative. Most currently available data are derived from relatively short follow‐up periods; thus, the long‐term biological behavior of transplanted cells and the durability of their clinical benefits remain to be fully elucidated. Consequently, extended longitudinal studies are required for a comprehensive assessment.

Notably, postmortem analyzes of patients more than a decade after fVM transplantation have revealed the presence of Lewy body pathology within a subset of grafted neurons (Kordower et al. 2008; Li et al. 2008, 2010, 2016; Kurowska et al. 2011). While the functional impact of this host‐to‐graft spread remains unclear, it is plausible that PSC‐derived grafts may also be susceptible to α‐synuclein pathology over time. These findings underscore that targeting α‐synuclein—the central pathogenic protein in PD—remains an important priority alongside cell‐based interventions. Accordingly, combinatorial strategies integrating cell therapy with interventions designed to suppress pathological progression represent an important direction for future research.

Furthermore, while transplantation therapy primarily targets motor symptoms, PD is characterized by a broad spectrum of non‐motor manifestations—including cognitive impairment, autonomic dysfunction, and sleep disturbances—which increasingly contribute to disease burden as the disease progresses. In the Kyoto trial, no significant improvement was observed in MDS‐UPDRS Part I scores, consistent with the understanding that non‐motor symptoms often require therapeutic approaches beyond dopaminergic replacement (Armstrong and Okun 2020).

## 7 Conclusion

Clinical trials of stem cell–based transplantation have overcome the historical limitations of fetal tissue approaches, opening new avenues for cell‐based therapies in PD. In particular, the demonstration of safety through high‐purity cell preparations, together with the feasibility of simplified immunosuppressive regimens, represents a major advance in the field.

However, several critical challenges remain, including the optimization of immune modulation, enhancement of graft survival, identification of patients most likely to benefit, and rigorous evaluation of long‐term safety and durability. Addressing these challenges will be essential for the further refinement of this therapeutic paradigm.

Ultimately, cell‐based therapies for PD hold the potential to become transformative interventions capable of substantially improving patients' QoL. The progress achieved to date represents a paradigm shift—moving beyond conventional symptomatic management toward a new era of regenerative medicine aimed at restoring neurological function.

## Author Contributions

**Etsuro Nakanishi:** conceptualization, writing – original draft, writing – review and editing. **Hodaka Yamakado:** conceptualization, writing – original draft, writing – review and editing. **Jun Takahashi:** conceptualization, writing – original draft, writing – review and editing. **Ryosuke Takahashi:** conceptualization, writing – review and editing, writing – original draft. **Nobukatsu Sawamoto:** conceptualization, writing – review and editing, writing – original draft.

## Funding

Jun Takahashi was supported by a grant from the Research Project for Practical Application of Regenerative Medicine of the Japan Agency for Medical Research and Development (AMED) [23bk0104126h0003].

## Conflicts of Interest

Ryosuke Takahashi receives research grants from Sumitomo Pharma. Nobukatsu Sawamoto and Jun Takahashi receive research grants from Sumitomo Pharma and RACTHERA Co. Ltd.

## Data Availability

The authors have nothing to report.
